# Association of Preprocedural SYNTAX Score With Outcomes in Impella-Assisted High-Risk Percutaneous Coronary Intervention

**DOI:** 10.1016/j.jscai.2024.101981

**Published:** 2024-04-17

**Authors:** Giorgio A. Medranda, Haroon A. Faraz, Julia B. Thompson, Yiran Zhang, Aditya S. Bharadwaj, Eric A. Osborn, Arsalan Abu-Much, Alexandra J. Lansky, Mir B. Basir, Jeffrey W. Moses, William W. O’Neill, Cindy L. Grines, Suzanne J. Baron

**Affiliations:** a Division of Cardiology, NYU Langone Hospital–Long Island, Mineola, New York; b Interventional Cardiology, Hackensack University Medical Center, Hackensack, New Jersey; c Clinical Trials Center, Cardiovascular Research Foundation, New York, New York; d Department of Cardiology, Loma Linda University Medical Center, Loma Linda, California; e Department of Medicine, Cardiovascular Division, Beth Israel Deaconess Medical Center, Boston, Massachusetts; f Section of Cardiovascular Medicine, Department of Internal Medicine, Yale School of Medicine, New Haven, Connecticut; g Division of Cardiology, Henry Ford Health System, Detroit, Michigan; h Division of Cardiology, NewYork-Presbyterian Hospital/Columbia University Irving Medical Center, New York, New York; i St. Francis Hospital & Heart Center, Roslyn, New York; j Department of Cardiology, Northside Hospital Cardiovascular Institute, Atlanta, Georgia; k Interventional Cardiovascular Research, Massachusetts General Hospital, Boston, Massachusetts; l Baim Institute for Clinical Research, Boston, Massachusetts

**Keywords:** high-risk percutaneous coronary intervention, major adverse cardiovascular and cerebrovascular events, outcomes, SYNTAX score

## Abstract

**Background:**

Patients with complex coronary artery disease, as defined by high SYNTAX scores, undergoing percutaneous coronary intervention (PCI) have poorer outcomes when compared with patients with lower SYNTAX I scores. This study aimed to assess if mechanical circulatory support using Impella mitigates the effect of the SYNTAX I score on outcomes after high-risk percutaneous coronary intervention (HRPCI).

**Methods:**

Using data from the PROTECT III study, patients undergoing Impella-assisted HRPCI between March 2017 and March 2020 were divided into 3 cohorts based on SYNTAX I score—low (≤22), intermediate (23-32), and high (≥33). Procedural and clinical outcomes out to 90 days were compared between groups. Multivariable regression analysis was used to assess the impact of SYNTAX I score on major adverse cardiovascular and cerebrovascular events (MACCE) at 90 days.

**Results:**

A total of 850 subjects with core laboratory–adjudicated SYNTAX I scores were identified (low: n = 310; intermediate: n = 256; high: n = 284). Patients with high SYNTAX I scores were older than those with low or intermediate SYNTAX I scores (72.7 vs 69.7 vs 70.1 years, respectively; *P* < .01). After adjustment for covariates, high SYNTAX I score remained a significant predictor of 90-day MACCE (hazard ratio [HR], 2.14; 95% CI, 1.42-3.69; *P* < .01 vs low), whereas intermediate SYNTAX I score was not (HR, 0.92; 95% CI, 0.47-1.77; *P* = .80 vs low). These findings persisted after adjustment for post-PCI SYNTAX I score.

**Conclusions:**

A high SYNTAX I score was associated with higher rates of 90-day MACCE in patients who underwent Impella-assisted HRPCI. Further research is needed to understand the patient and procedural factors driving this finding.

## Introduction

Despite many advancements in the field of interventional cardiology over the last decade, there remains a substantial group of patients in whom percutaneous coronary intervention (PCI) is considered high risk.^1^^,^^2^ High-risk percutaneous coronary intervention (HRPCI) often includes a combination of clinical variables (decreased left ventricular ejection fraction [LVEF], advanced age, and valvular disease) and procedural characteristics (high anatomical complexity and anticipated need for atherectomy).^1^^,^^3^^,^^4^ The Synergy Between Percutaneous Coronary Intervention with TAXUS and Cardiac Surgery (SYNTAX) trial developed a scoring system to standardize the grading of complexity of coronary artery disease (CAD) and demonstrated that patients with the most complex CAD, as defined by SYNTAX I scores of ≥33, had increased rates of major adverse cardiac and cerebrovascular events (MACCE) after PCI when compared with those with less complex CAD.^5^ Multiple studies have since validated the association between high SYNTAX I scores and MACCE after PCI.^6^, ^7^, ^8^ Mechanical circulatory support (MCS) devices, including the intra-aortic balloon pump and Impella (Abiomed) percutaneous left ventricular assist devices, have been utilized as methods to mitigate the associated risk and improve hemodynamic measures during HRPCI.^9^, ^10^, ^11^, ^12^, ^13^ Accordingly, the United States Food and Drug Administration (FDA) expanded the indication for the use of Impella devices to support patients with complex CAD with and without depressed left ventricular function undergoing HRPCI in 2018.^14^ Whether MCS using the Impella device mitigates the effect of the preprocedural SYNTAX I score on HRPCI outcomes remains unknown. To address this gap in knowledge, we sought to leverage data from the contemporary PROTECT III study to evaluate and compare short-term outcomes in patients undergoing HRPCI, stratified by SYNTAX I score.

## Methods

### Study population and registry conduct

The global catheter-based ventricular assist device (cVAD) registry (NCT04136392) and PROTECT III study design and preliminary clinical results have been described previously.^15^^,^^16^ In short, the PROTECT III study was a multicenter, single-arm, observational, FDA-audited, postapproval study that sought to evaluate the postmarket safety and efficacy of the Impella 2.5 and Impella CP devices for use during HRPCI in hemodynamically stable patients with severe CAD at 46 centers in the United States. Patients were enrolled in the study once an operator deemed the procedure high risk and made the decision to proceed with Impella insertion either before or during PCI for the purpose of preventing hemodynamic instability during the procedure. Patients requiring bailout Impella treatment or patients with ongoing cardiogenic shock were excluded. The PCI and postprocedural care of patients was performed according to the treating physician’s standard of care.

Baseline characteristics, preprocedural echocardiographic parameters, baseline laboratory values, and procedural characteristics were collected during the index hospitalization. Patients were followed up through 90 days postprocedure for adverse events. An angiographic core laboratory (Beth Israel Deaconess Medical Center, Boston, Massachusetts) was used for calculation of the pre-PCI and post-PCI SYNTAX I scores. The study cohort included all patients in the PROTECT III study with core laboratory–adjudicated SYNTAX I scores. Patients were subsequently divided into 3 cohorts—those with high pre-PCI SYNTAX I scores (≥33), those with intermediate pre-PCI SYNTAX I scores (23-32), and those with low pre-PCI SYNTAX I scores (≤22). These cutoffs were chosen to be consistent with previously published literature demonstrating the clinical significance of these thresholds in the pivotal SYNTAX trial.^5^^,^^17^ The study was conducted in accordance with the tenets of the Declaration of Helsinki. The research protocols for the cVAD registry were approved by the relevant institutional review boards at each participating center. An independent 12-member steering committee, including interventional cardiologists, cardiac surgeons, and heart failure specialists, oversaw the conduct of the cVAD registry. The sponsor (Abiomed) oversaw study data management and source document verification and provided funding to Cardiovascular Research Foundation (New York, New York) for statistical analysis.

### Study end points

The primary end point for this analysis was MACCE at 90 days, defined as the composite of all-cause death, myocardial infarction (MI), stroke/transient ischemic attack, and repeat revascularization. Secondary end points included 1-year mortality, MACCE at discharge and 30 days, as well as the individual components of MACCE at all time points through 90 days. All MACCE components were adjudicated by an independent clinical events committee.

### Statistical analysis

Data were presented as mean ± SD for continuous variables and as number (percentage) for categorical variables. Continuous variables were compared using the 1-way ANOVA test or Kruskal–Wallis test if normality assumption was not met, and categorical variables were compared using the χ^2^ or Fisher exact test when appropriate. Multivariate Cox regression analysis was used to assess the impact of pre-PCI SYNTAX I score on MACCE rates at 90 days. Covariates included in the model were age, sex, body mass index, diabetes, tobacco use, peripheral arterial disease, chronic pulmonary disease, prior stroke, prior PCI, prior coronary artery bypass graft surgery, valvular heart disease, acute coronary syndrome as indication for PCI, renal insufficiency, LVEF (by increments of 5%), and post-PCI SYNTAX I score. Imputation of the mean value within each cohort was used to account for missing data in the LVEF variables. A *P* value <.05 was considered statistically significant. All analyses were done using SAS version 9.4 (SAS Institute).

## Results

A total of 1237 patients were enrolled in the PROTECT III study between March 2017 and March 2020. Of these patients, 850 subjects had core laboratory–adjudicated SYNTAX I scores (low: n = 310; intermediate: n = 256; high: n = 284) and, thus, were included in the analysis (Supplemental Figure S1). Baseline clinical characteristics of the cohorts are summarized in Table 1. In general, patients in the high SYNTAX I score cohort were older (high: 72.7 ± 10.4 years; vs intermediate: 70.1 ± 11.1 years; vs low: 69.7 ± 11.5 years; *P* < .01). Mean LVEF was similar across all cohorts (32.7% in the low SYNTAX I score cohort, 33.6% in the intermediate SYNTAX I score cohort, and 35.7% in the high SYNTAX I score cohort; *P* = .10). There were no significant differences between other baseline clinical characteristics between the cohorts (Table 1).

**Table 1:** Baseline characteristics among SYNTAX I score cohorts.

	Low (n = 310)	Intermediate (n = 256)	High (n = 284)	*P*
Age, y	69.7 ± 11.5	70.1 ± 11.1	72.7 ± 10.4	<.01
Male sex	236/310 (76.1)	180/256 (70.3)	199/284 (70.1)	.18
White or Caucasian	203/310 (65.5)	164/256 (70.3)	181/284 (70.1)	.89
Body mass index, kg/m^2^	29.1 ± 7.0	28.9 ± 7.0	28.5 ± 5.9	.60
Hypertension	281/309 (90.9)	232/255 (91.0)	257/281 (91.5)	.97
Hyperlipidemia	239/308 (77.6)	196/255 (76.9)	221/283 (78.1)	.94
Diabetes mellitus	170/308 (55.2)	137/255 (53.7)	161/283 (56.9)	.76
Renal insufficiency	92/306 (30.1)	73/255 (28.6)	104/282 (36.9)	.08
Tobacco use	196/304 (64.5)	153/250 (61.2)	160/273 (58.6)	.35
Prior CVA/TIA	48/308 (15.6)	42/254 (16.5)	53/281 (18.9)	.56
Prior MI	116/303 (38.3)	104/244 (42.6)	118/270 (43.7)	.38
Prior PCI	111/307 (36.2)	102/256 (39.8)	106/279 (38.0)	.67
Prior CABG	36/310 (11.6)	22/254 (8.7)	31/282 (11.0)	.50
Cardiomyopathy	115/278 (41.4)	88/226 (38.9)	94/243 (38.7)	.79
LVEF, %	32.7 ± 15.3	33.6 ± 14.7	35.7 ± 15.5	.10
Valvular heart disease	63/288 (21.9)	34/224 (15.2)	43/248 (17.3)	.13
Arrhythmia	84/287 (29.3)	48/227 (21.1)	67/247 (27.1)	.10
Chronic lung disease	70/308 (22.7)	66/252 (26.2)	59/282 (20.9)	.35
Peripheral artery disease	64/308 (20.8)	47/256 (18.4)	67/277 (24.2)	.25

Values are mean ± SD or n/N (%).

CABG, coronary artery bypass graft; CVA, cerebrovascular accident; LVEF, left ventricular ejection fraction; MI, myocardial infarction; SYNTAX, Synergy Between Percutaneous Coronary Intervention with TAXUS and Cardiac Surgery; PCI, percutaneous coronary intervention; SYNTAX, Synergy Between Percutaneous Coronary Intervention with TAXUS and Cardiac Surgery; TIA, transient ischemic attack.

From a procedural standpoint, patients had a similar likelihood of undergoing PCI for management of acute coronary syndrome (59.3% vs 59.9% vs 62.9; *P* = .67) (Table 2). Left main coronary artery lesions were treated significantly more in patients with a high SYNTAX I score (64.7% vs 51.6% [intermediate] vs 27.4% [low]; *P* < .01) (Table 2). Atherectomy was also used more frequently in patients with a high SYNTAX I score as well (52.0% vs 35.7% [intermediate] vs 35.5% [low]; *P* < .01). No significant difference was noted in the median duration of Impella treatment between the cohorts (5.52 vs 5.10 vs 6.36 hours; *P* = .67), although the Impella CP device was used more frequently in the high SYNTAX I score group. There was no difference in PCI-related complications between the cohorts (4.3% vs 3.9% vs 5.3%; *P* = .76).

**Table 2:** Procedural details among SYNTAX I score cohorts.

	Low (n = 310)	Intermediate (n = 256)	High (n = 284)	*P*
ACS presentation	162/273 (59.3)	133/222 (59.9)	158/251 (62.9)	.67
Lesion location				
Left main	85/310 (27.4)	132/256 (51.6)	183/283 (64.7)	<.01
Left anterior descending	213/310 (68.7)	191/256 (74.6)	201/283 (71.0)	.30
Left circumflex	110/310 (35.5)	123/256 (48.0)	152/283 (53.7)	<.01
Right coronary artery	95/310 (30.6)	82/256 (32.0)	63/283 (22.3)	.02
Ramus	10/310 (3.2)	17/256 (6.6)	33/283 (11.7)	<.01
Graft	8/310 (2.6)	10/256 (3.9)	8/283 (2.8)	.63
Preprocedural hemodynamics				
Systolic blood pressure, mm Hg	126.8 ± 22.9	125.7 ± 23.3	127.6 ± 22.5	.64
Diastolic blood pressure, mm Hg	71.8 ± 14.7	69.9 ± 13.9	69.8 ± 12.5	.14
Cardiac output, L/min	4.3 [3.6, 5.1]	4.7 [3.7, 5.1]	4.4 [3.6, 5.5]	.92
Cardiac index, L/min/m^2^	2.4 [1.9, 2.8]	2.3 [1.9, 2.5]	2.3 [1.9, 2.8]	.71
Impella CP use	194/310 (62.6)	176/256 (69.0)	208/284 (73.2)	.02
Impella duration, h	5.52 ± 16.9	5.10 ± 13.0	6.36 ± 16.3	.67
Contrast used, mL	180 [120, 260]	176 [125, 250]	199 [135, 275]	.13
Atherectomy	108/304 (35.5)	91/255 (35.7)	144/277 (52.0)	<.01
Transfemoral access	292/310 (94.2)	243/256 (94.9)	261/284 (91.9)	.32
Vascular closure device	245/310 (84.5)	208/256 (81.3)	213/284 (75.0)	.05
Pre-PCI SYNTAX score	15.6 ± 4.5	27.4 ± 2.8	42.1 ± 8.2	<.01
Post-PCI SYNTAX score	2.8 ± 3.7	5.5 ± 6.9	11.9 ± 10.2	<.01
Change in SYNTAX score	−12.8 ± 4.7	−21.9 ± 7.0	−30.2 ± 10.6	<.01

Values are mean ± SD, median [Q1, Q3], or n/N (%). ACS included STEMI, NSTEMI, and unstable angina.

ACS, acute coronary syndrome; NSTEMI, non–ST segment–elevated myocardial infarction; PCI, percutaneous coronary intervention; RCA, right coronary artery; STEMI, ST segment–elevated myocardial infarction; SYNTAX, Synergy Between Percutaneous Coronary Intervention with TAXUS and Cardiac Surgery.

In unadjusted analyses, 30-day and 90-day MACCE rates were significantly more frequent in the high SYNTAX I cohort (8.0% [low] vs 6.1% [intermediate] vs 13.0% [high]; *P* = .02, and 10.1% [low] vs 9.1% [intermediate] vs 19.0% [high]; *P* < .01, respectively), and there was a trend toward higher in-hospital MACCE rates as well (5.2% vs 3.1% vs 7.7%; *P* = .06) (Table 3 and Central Illustration). In general, MACCE rates were driven by higher rates of cardiovascular disease–related mortality at all time points in the high SYNTAX I cohort, although cardiovascular disease–related mortality was only statistically significantly different between the cohorts at the 90-day time point. The unadjusted all-cause mortality at 1 year was also higher in patients with high pre-PCI SYNTAX I scores (15.5% [low] vs 18.4% [intermediate] vs 28.5% [high]; *P* < .01). No significant difference in cerebrovascular events, MI, or revascularization rates were noted between the cohorts at any time point over 90 days (Table 3). When type of cardiovascular death at 90 days was examined among the cohorts, there was a significantly higher occurrence of death related to PCI-procedural complications and death related to decompensated heart failure with multisystem organ failure in the high SYNTAX I cohort (Supplemental Table S1). Review of site-reported adverse events at discharge demonstrated a greater occurrence of stage 2 or 3 renal injury in patients with a high SYNTAX I score although mean creatinine concentration at the time of procedure was not different between the 3 groups (mean, 1.2 mg/dL in all groups; *P* = .94) (Supplemental Table S2). A trend toward higher rates of significant bleeding in patients with a high SYNTAX I score was also noted (1.3% vs 2.4% vs 4.2%; *P* = .08) despite similar rates of vascular closure device use as compared with patients with intermediate and low SYNTAX I scores (Supplemental Table S2).

**Table 3:** Unadjusted clinical outcomes among SYNTAX I score cohorts.

	Low (n = 310)	Intermediate (n = 256)	High (n = 284)	*P*
In-hospital outcomes^a^				
MACCE	16/310 (5.2)	8/256 (3.1)	22/284 (7.7)	.06
All-cause mortality	11/310 (3.5)	7/256 (2.7)	19/284 (6.7)	.06
CV-related mortality	11/310 (3.5)	7/256 (2.7)	17/284 (6.0)	.13
Non–CV-related mortality	0	0	2/284 (0.7)	.14
Myocardial infarction	4/310 (1.3)	2/256 (0.8)	2/284 (0.7)	.72
Stroke/TIA	3/310 (1.0)	3/256 (1.2)	5/284 (1.8)	.68
Revascularization	2/310 (0.6)	0	0	.17
30-Day outcomes^a^				
MACCE	8.0 (22)	6.1 (14)	13.0 (33)	.02
All-cause mortality	6.8 (18)	4.9 (11)	11.3 (28)	.02
CV-related mortality	6.4 (17)	4.9 (11)	10.1 (25)	.07
Non–CV-related mortality	0.4 (1)	0	1.3 (3)	.16
Myocardial infarction	2.2 (6)	2.3 (5)	2.5 (6)	.98
Stroke/TIA	1.0 (3)	1.2 (3)	2.7 (7)	.28
Revascularization	0.7 (2)	0.9 (2)	0.9 (2)	.99
90-Day outcomes^a^				
MACCE	10.1 (27)	9.1 (20)	19.0 (46)	<.01
All-cause mortality	8.5 (22)	5.9 (13)	16.1 (39)	<.01
CV-related mortality	7.7 (20)	5.9 (13)	14.3 (34)	<.01
Non–CV-related mortality	0.9 (2)	0	2.3 (5)	.06
Myocardial infarction	3.1 (8)	3.3 (7)	5.6 (12)	.42
Stroke/TIA	1.0 (3)	1.2 (3)	3.2 (8)	.16
Revascularization	2.1 (5)	2.5 (5)	3.0 (6)	.88
1-Year outcomes^a^				
All-cause mortality	15.5 (38)	18.4 (36)	28.5 (65)	<.01

In-hospital outcomes are presented as the proportion of patients with events and represent n/N (%). The 30-day, 90-day, and 1-year outcomes are presented as the Kaplan-Meier event rates and represent % (n patients with event).

CV, cardiovascular; MACCE, major adverse cardiovascular and cerebrovascular event; SYNTAX, Synergy Between Percutaneous Coronary Intervention with TAXUS and Cardiac Surgery; TIA, transient ischemic attack.

a Patients might have experienced more than 1 component of MACCE but were counted only within the composite end point of MACCE once at the time of first event to occur.

**Central Illustration:**
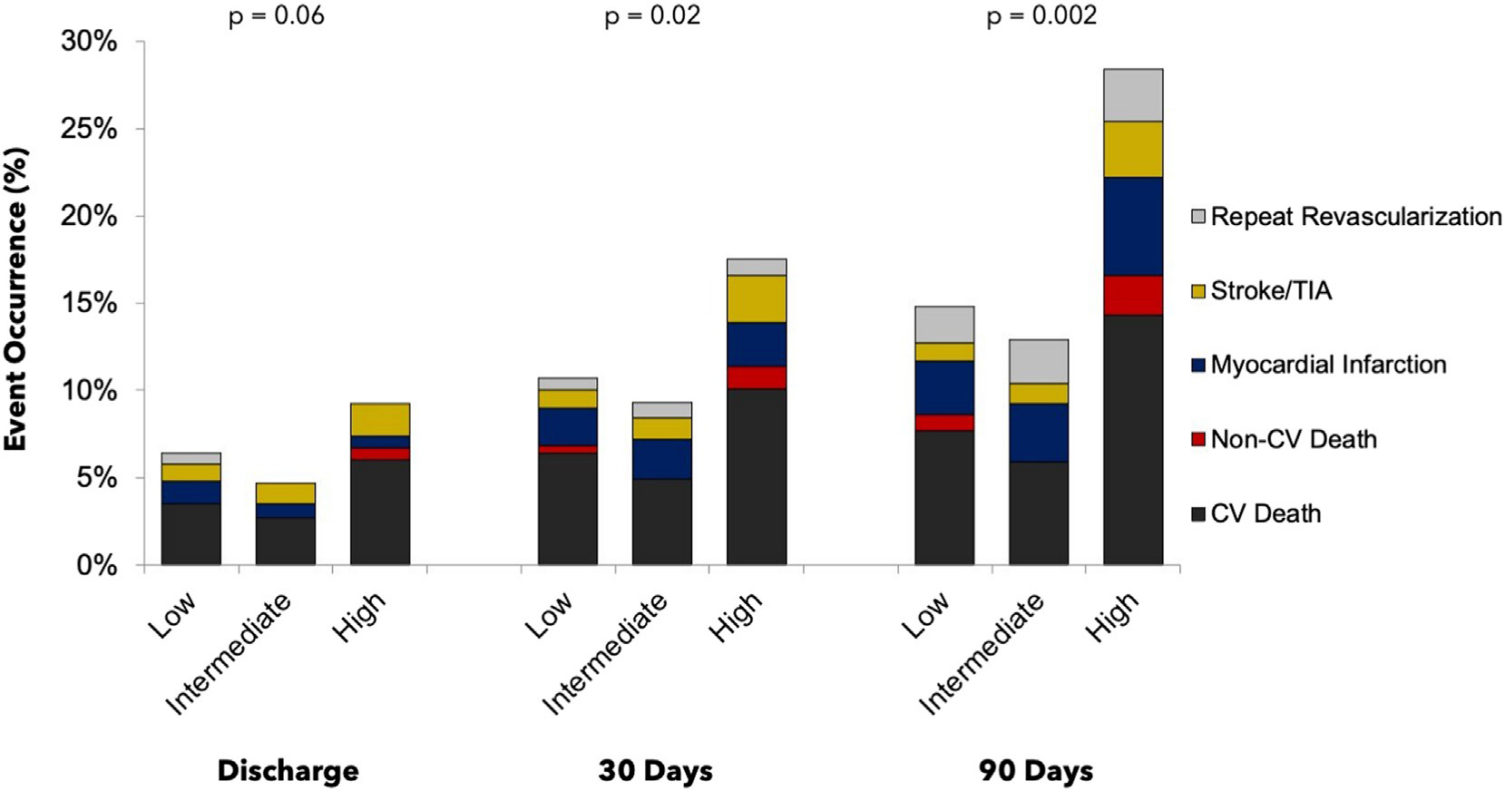
Major cardiac and cerebrovascular outcomes stratified by pre-PCI SYNTAX I scores at discharge, 30 days, and 90 days. Individual components of the composite MACCE outcome (cardiovascular death, noncardiovascular death, repeat revascularization, stroke/TIA, and myocardial infarction) are shown here stratified by pre-PCI SYTNAX I scores. As patients could have experienced multiple individual components of the composite MACCE outcome, the overall rates of the composite of MACCE are lower than the sum of the individual components. CV, cardiovascular; MACCE, major adverse cardiovascular and cerebrovascular event; PCI, percutaneous coronary intervention; SYNTAX, Synergy Between Percutaneous Coronary Intervention with TAXUS and Cardiac Surgery; TIA, transient ischemic attack.

In multivariable analyses, a high pre-PCI SYNTAX I score was found to be associated with an increased risk for MACCE at 90 days (HR, 2.14; 95% CI, 1.42-3.69; *P* < .01), as well as increased 1-year mortality (HR, 1.99; 95% CI, 1.25-3.16; *P* < .01); however, no effect was seen with an intermediate pre-PCI SYNTAX I score when compared to patients with a low pre-PCI SYNTAX I score (Table 4). This association persisted even when post-PCI SYNTAX I score was included in the model either as a continuous variable or as a dichotomous variable (<8 vs ≥8) for both 90-day MACCE rates and 1-year mortality (Table 4).

**Table 4:** Effect of pre-PCI SYNTAX I score on adjusted clinical outcomes.

	90-Day MACCE		1-Year mortality	
	Hazard ratio (95% CI)	*P*	Hazard ratio (95% CI)	*P*
Without post-PCI SYNTAX I score				
Intermediate vs low SYNTAX I score	0.92 (0.47-1.77)	.80	1.19 (0.71-2.01)	.52
High vs low SYNTAX I score	2.14 (1.42-3.69)	<.01	1.99 (1.25-3.16)	<.01
With post-PCI SYNTAX I score as continuous variable				
Intermediate vs low SYNTAX I score	0.92 (0.47-1.79)	.80	1.16 (0.68-1.98)	.59
High vs low SYNTAX I score	2.06 (1.11-3.84)	<.01	1.80 (1.06-3.06)	.03
With post-PCI SYNTAX I score as dichotomous variable <8 or ≥8				
Intermediate vs low SYNTAX I score	0.91 (0.47-1.78)	.79	1.20 (0.70-2.03)	.51
High vs low SYNTAX I score	2.04 (1.11-3.73)	<.01	1.95 (1.17-3.25)	.01

MACCE defined as the composite of all-cause death, myocardial infarction, stroke/transient ischemic attack, and any repeat revascularization.

MACCE, major adverse cardiovascular and cerebrovascular event; PCI, percutaneous coronary intervention; SYNTAX, Synergy Between Percutaneous Coronary Intervention with TAXUS and Cardiac Surgery.

## Discussion

This post hoc analysis of the PROTECT III study is the first to compare clinical outcomes out to 90 days across the full spectrum of SYNTAX I scores in patients undergoing Impella-assisted HRPCI. The principal findings of this analysis were as follows: (1) patients with high SYNTAX I scores were found to have numerically higher rates of MACCE at discharge and statistically significantly higher rates of MACCE at 30 days and 90 days postprocedurally; and (2) high SYNTAX I score remained significantly associated with 90-day MACCE rates and 1-year mortality even after adjustment for multiple covariates, including post-PCI SYNTAX I score.

In comparing MACCE rates observed in this analysis with those observed within the pivotal SYNTAX trial,^5^ we found substantially higher MACCE rates at 30 and 90 days in patients in all 3 tiers of SYNTAX I scores. There are several possibilities for this finding. First, the patients included in the PROTECT III study were certainly a higher risk and sicker patient population than those included in the SYNTAX trial as demonstrated by the older age and substantially higher rates of comorbidities in our patient population. Furthermore, the SYNTAX trial excluded those with severely reduced LVEF as well as those at prohibitive-risk for surgical revascularization. Accordingly, it would be expected that our study population would be at a greater risk for MACCE. Second, all patients included in the PROTECT III study were deemed to need MCS in the form of an Impella device, thereby suggesting that these patients were high risk for the procedure; however, because the decision to implement Impella was at the discretion of the PROTECT III operator, there may be heterogeneity with regards to each operators’ threshold for deeming a patient high risk. Although the use of MCS was not formally reported in the SYNTAX trial, the rate is presumed to have been very low (as it was not included in a detailed microcosting analysis of the trial) and was likely limited to intra-aortic balloon pumps only since the Impella device was not commercially available during the time that the SYNTAX trial enrolled.^18^ Finally, it is likely that the PCIs performed in the PROTECT III study were more complex than the procedures performed in the SYNTAX trial, as suggested by the high rates of atherectomy (60.8% in our overall study cohort) as compared with an atherectomy rate of <15% in the SYNTAX trial, thereby further suggesting that the patients of the SYNTAX trial may not be completely comparable with the types of patients who are routinely treated with Impella support during PCI in the current era.^18^

Despite the difference in patient populations between the SYNTAX trial and other current PCI trials, the SYNTAX I score has been shown to be valuable in predicting prognosis after PCI in a number of analyses, with high SYNTAX I scores consistently being associated with worse short-term and long-term clinical outcomes.^19^, ^20^, ^21^, ^22^, ^23^ Similarly, in our study, patients with a high SYNTAX I score had substantially higher rates of MACCE, specifically cardiovascular disease–related death, through 90 days. In examining the specific causes of cardiovascular disease–related death, high SYNTAX I score patients were more likely to die of heart failure with multiorgan failure or PCI-related complications. Certainly, this finding could be related to the presence of certain comorbidities, such as prior stroke and peripheral arterial disease, which were numerically higher in the high SYNTAX I cohort. In fact, several studies have demonstrated that the SYNTAX I score can be a surrogate marker of clinical comorbidity, due to concomitant cardiovascular disease states and associated end-organ damage, in addition to anatomic complexity.^24^, ^25^, ^26^, ^27^, ^28^, ^29^, ^30^ Additionally, certain comorbidities may be exacerbated in the setting of complex PCI. For example, although there was no difference in baseline creatinine at the time of the procedure, there was a notable trend toward a higher incidence of renal insufficiency at baseline, and this may have translated to the greater rates of stage 2 or 3 renal dysfunction that was observed postprocedurally in this cohort. Several studies have demonstrated that acute kidney injury post-PCI has been found to be strongly associated with in-hospital mortality.^31^ Moreover, there was a nonsignificant trend toward higher rates of major bleeding postprocedurally in the high SYNTAX I cohort, which may be related to a combination of factors including older age and more frequent use of the Impella CP device (which utilizes a 14F sheath as opposed to a 13F sheath). Multiple studies have demonstrated that PCI-related bleeding is associated with increased in-hospital mortality and 1 year mortality rates.^32^^,^^33^ Taken together, the correlation of a high SYNTAX I score with specific comorbidities may account for the increased rates of MACCE in this group.

Recent data have suggested that the SYNTAX I score calculated after a PCI is complete (known as the residual SYNTAX I score) may be just as important as a pre-PCI SYNTAX I score.^34^^,^^35^ Indeed, researchers have found that both 5-year and 10-year mortality rates are significantly higher in patients with a residual SYNTAX I score of >8, even after adjusting for other anatomical and clinical factors including pre-PCI SYNTAX I score (10 years: adjusted HR, 3.40; 95% CI, 2.13-5.43).^34^^,^^35^ It has been hypothesized that this finding is related to the increasingly frequent observation that incomplete revascularization is associated with poorer clinical outcomes in patients who underwent PCI presenting with both stable and unstable CAD.^36^, ^37^, ^38^, ^39^, ^40^, ^41^, ^42^ One of the proposed benefits of Impella-assisted PCI is that it may enable operators to achieve complete revascularization and, thus, lower post-PCI residual SYNTAX I scores. Indeed, the extent of revascularization observed in this analysis was large, particularly in the high SYNTAX I score cohort in which the mean change in SYNTAX I score was 30.2. Nevertheless, our analysis found that a high pre-PCI SYNTAX I score was still significantly associated with poor outcomes at 90 days even after controlling for post-PCI residual SYNTAX I score. This observation could be secondary to the fact that the majority of the effect of incomplete revascularization is realized at time points beyond 90 days, although high pre-PCI SYNTAX I scores remained significantly associated with 1-year mortality in adjusted analyses. It is also possible that it is the functional significance of a residual coronary lesion as opposed to a purely anatomical description that is the true mediator of poor outcomes. Indeed, Kobayashi et al demonstrated that residual angiographic disease as defined by the SYNTAX I score was not associated with MACCE after fractional-flow-reserve guided complete revascularization.^43^ As such, if the majority of PROTECT III patients achieved complete functional revascularization, then it would follow that the calculated post-PCI residual SYNTAX I score would have minimal effect on outcomes.

### Limitations

This study has several limitations. First, despite our multivariate analysis, it is certainly possible that there remain unmeasured confounders, which may affect the conclusions drawn herein. Second, some of the variables had up to ∼19% missing data. Although missing data were managed with multiple imputation, possible that missing data could have skewed our findings. That said, when the regression models were rerun without LVEF included (as this was the variable with the most missing data—19.3%), the results were very similar (Supplemental Table S3). Third, as a single-arm study, the PROTECT III study did not include a comparator arm of patients undergoing high-risk PCI without use of Impella, limiting our ability to report the relative reduction of risk that the Impella device theoretically offered to this patient populations. Additionally, due to the lack of granularity of certain data variables, we were unable to attribute differences in PCI-related complications to the Impella device or to the procedure itself; we were also unable to delineate the type of recurrent MI that occurred. Furthermore, there was a proportion of patients included in the PROTECT III study without sufficient angiographic data to allow for precise core laboratory–adjudicated SYNTAX I score calculation. These patients were excluded from our analysis, which could affect the generalizability of our findings. Finally, our follow-up MACCE data was limited to 90 days, thus the effect of pre-PCI SYNTAX I and post-PCI SYNTAX I scores on MACCE rates beyond 90 days remains unknown and will be important to investigate in future studies.

## Conclusions

Despite aggressive revascularization, a high SYNTAX I score was associated with higher rates of 90-day MACCE and 1-year mortality rates in patients who underwent Impella-assisted HRPCI. Further research is needed to understand the specific patient and procedural factors driving this finding.
