# Supplementary material for: Association of Preprocedural SYNTAX Score With Outcomes in Impella-Assisted High-Risk Percutaneous Coronary Intervention
Source: J Soc Cardiovasc Angiogr Interv. 2024 Apr 17;3(8):101981. doi: 10.1016/j.jscai.2024.101981 (PMC11330901; doi:10.1016/j.jscai.2024.101981)
Supplement: Supplemental Figure S1 and Supplemental Tables S1-S3 [file mmc1.docx]

Supplemental Appendix

**Supplemental Figure S1. Study Flow Chart.
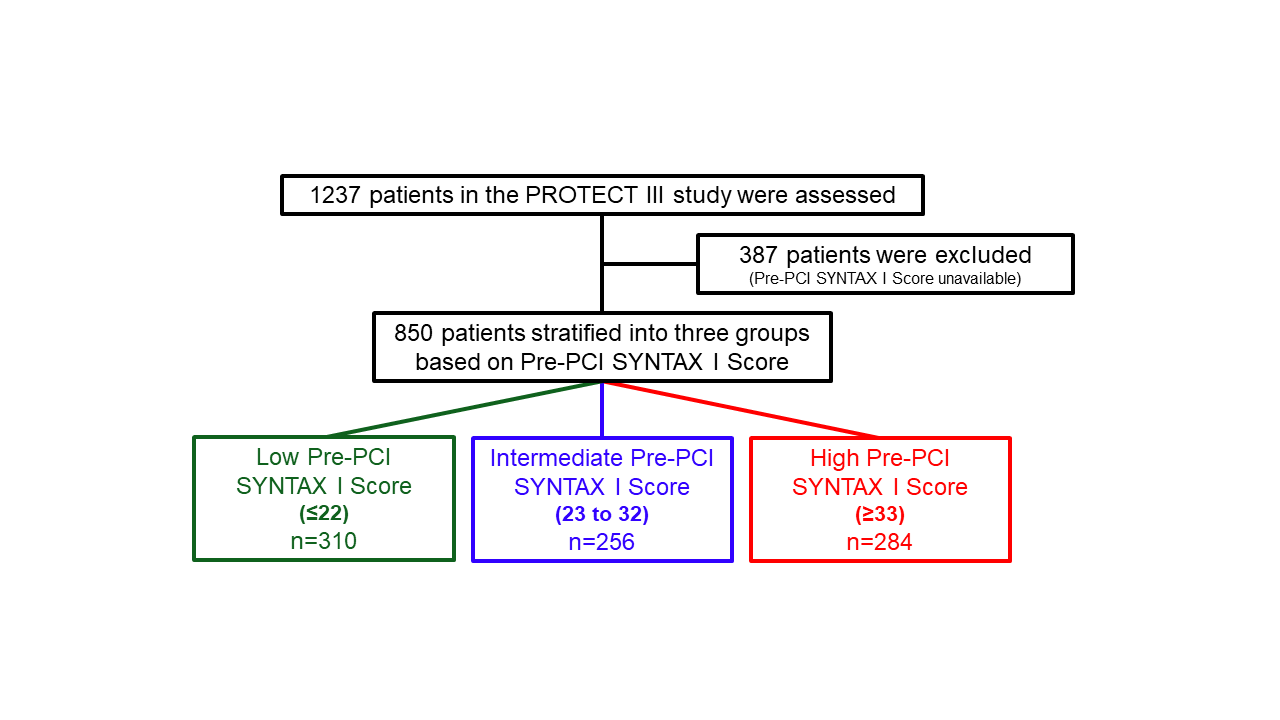
**

**Supplemental Table S1: Type of Cardiovascular Death at 90 Days in Each SYNTAX Score Cohort**

|  | **Low**  **(n=310)** | **Intermediate**  **(n=256)** | **High**  **(n=284)** | **P-value** |
| --- | --- | --- | --- | --- |
| MI-related | 3.2% (8) | 2.3% (5) | 2.9% (7) | 0.83 |
| Sudden Cardiac Death | 0.8% (2) | 0.5% (1) | 2.1% (4) | 0.33 |
| Heart Failure/Cardiogenic Shock | 0 | 0 | 0 | N/A |
| CVA-related | 0.4% (1) | 0.5% (1) | 1.3% (3) | 0.43 |
| Decompensated Heart Failure with Multisystem Organ Failure | 0.4% (1) | 0 | 2.5% (5) | 0.02 |
| PCI-procedural Complications | 1.0% (3) | 0.8% (2) | 4.2% (10) | 0.02 |
| Arrhythmia | 0 | 0.5% (1) | 0 | 0.34 |
| Other | 2.1% (5) | 1.5% (3) | 2.2% (5) | 0.79 |
| Values are % (n)  Abbreviations: CVA – cerebrovascular accident; MI – myocardial infarction; PCI – percutaneous coronary intervention; SYNTAX – Synergy Between Percutaneous Coronary Intervention with TAXUS and Cardiac Surgery | | | | |

**Supplemental Table S2: Site-Reported Adverse Events at Discharge in Each SYNTAX Score Cohort**

|  | **Low**  **(n=310)** | **Intermediate**  **(n=256)** | **High**  **(n=284)** | **P-value** |
| --- | --- | --- | --- | --- |
| Any Adverse Event | 25.2% (78) | 27.5% (70) | 33.1% (94) | 0.09 |
| Failure to Achieve Angiographic Success | 0.6% (2) | 0.4% (1) | 0.4% (1) | 0.85 |
| Pericardial Effusion requiring Drainage | 1.6% (5) | 0 | 2.1% (6) | 0.08 |
| Cardiac Arrest | 0.3% (1) | 2.7% (7) | 3.2% (9) | 0.03 |
| Cardiogenic Shock | 1.6% (5) | 1.2% (3) | 3.5% (10) | 0.12 |
| Acute Renal Dysfunction (Stage 2 or 3) | 2.6% (8) | 3.9% (10) | 7.4% (21) | 0.02 |
| Life-threatening, disabling, or major bleeding | 1.3% (4) | 2.4% (6) | 4.2% (12) | 0.08 |
| Anemia requiring transfusion | 6.1% (19) | 9.4% (24) | 7.0% (20) | 0.32 |
| Hematoma | 6.8% (21) | 5.5% (14) | 11.3% (32) | 0.03 |
| Vascular complication without surgery | 1.6% (5) | 1.2% (3) | 1.4% (4) | 0.91 |
| Vascular complication requiring surgery | 1.0% (3) | 0.8% (2) | 0.7% (2) | 0.94 |
| Limb Ischemia | 1.3% (4) | 1.6% (4) | 2.5% (7) | 0.53 |
| Infection | 1.6% (5) | 2.0% (5) | 4.2% (12) | 0.10 |
| Respiratory Dysfunction/failure | 1.0% (3) | 2.0% (5) | 2.5% (7) | 0.37 |
| Values are % (n)  Abbreviation: SYNTAX – Synergy Between Percutaneous Coronary Intervention with TAXUS and Cardiac Surgery | | | | |

**Supplemental Table S3: Effect of Pre-PCI SYNTAX Score on Adjusted 90-Day MACCE Rates Without Left Ventricular Ejection Fraction as CoVariate**

|  | **Hazard Ratio (95% CI)** | **P Value** |
| --- | --- | --- |
| **Without Post-PCI SYNTAX Score** | | |
| Intermediate vs. low SYNTAX score | 0.90 (0.47, 1.74) | 0.76 |
| High vs. low SYNTAX score | 1.99 (1.16, 3.42) | 0.01 |
| **With Post-PCI SYNTAX Score as Continuous Variable** | | |
| Intermediate vs. low SYNTAX score | 0.89 (0.46, 1.72) | 0.72 |
| High vs. low SYNTAX score | 1.82 (0.98, 3.37) | 0.06 |
| **With Post-PCI SYNTAX Score as Dichotomous Variable <8 or ≥8** | | |
| Intermediate vs. low SYNTAX score | 0.89 (0.46, 1.72) | 0.72 |
| High vs. low SYNTAX score | 1.83 (1.01, 3.32) | 0.05 |
| MACCE defined as the composite of all-cause death, myocardial infarction, stroke/transient ischemic attack, and any repeat revascularization.  Abbreviations: MACCE – major adverse cardiovascular and cerebrovascular events; PCI – percutaneous coronary intervention; SYNTAX – Synergy Between Percutaneous Coronary Intervention with TAXUS and Cardiac Surgery | | |
